# 7,9-Diallyl-6-methyl-7*H*-1,2,4-triazolo[4,3-*b*][1,2,4]triazepin-8(9*H*)-one

**DOI:** 10.1107/S160053680903133X

**Published:** 2009-08-15

**Authors:** Redwan Mohamed Zemama, Ibtissam Amari, Rachid Bouhfid, El Mokhtar Essassi, Seik Weng Ng

**Affiliations:** a Laboratoire de Chimie Organique Hétérocyclique, Pôle de compétences Pharmacochimie, Université Mohammed V-Agdal, BP 1014 Avenue Ibn Batout, Rabat, Morocco; b Institute of Nanomaterials and Nanotechnology, Avenue de l’Armée Royale, Madinat El Irfane, 10100 Rabat, Morocco; c Department of Chemistry, University of Malaya, 50603 Kuala Lumpur, Malaysia

## Abstract

The title compound, C_12_H_15_N_5_O, features a triazolyl ring fused with a seven-membered triazepinyl ring; the latter ring adopts a boat conformation with the allyl-bearing C atom as the prow and the C and N fused-ring atoms as the stern.

## Related literature

Triazepines are used in the treatment of neuronal disorders. They are also the reacta­nts for the synthesis of other heterocyclic compounds; see, for example: Essassi *et al.* (1977 ▶); Richter & Sheefelot (1991 ▶).
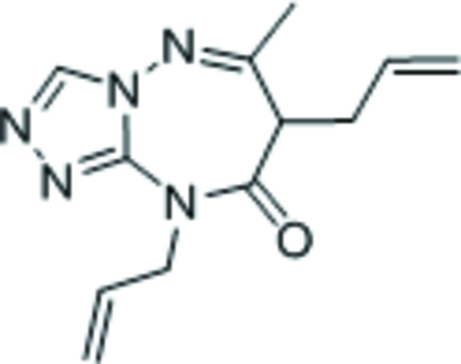


## Experimental

### 

#### Crystal data

C_12_H_15_N_5_O
*M* *_r_* = 245.29
Monoclinic, 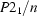

*a* = 7.4674 (3) Å
*b* = 8.3398 (3) Å
*c* = 20.2214 (6) Å
β = 95.174 (2)°
*V* = 1254.19 (8) Å^3^
*Z* = 4
Mo *K*α radiation
μ = 0.09 mm^−1^
*T* = 293 K
0.3 × 0.3 × 0.3 mm

#### Data collection

Bruker APEX2 diffractometer
Absorption correction: none
11394 measured reflections
2435 independent reflections
1600 reflections with *I* > 2σ(*I*)
*R*_int_ = 0.041

#### Refinement

*R*[*F* ^2^ > 2σ(*F* ^2^)] = 0.053
*wR*(*F* ^2^) = 0.176
*S* = 1.03
2435 reflections
164 parameters
H-atom parameters constrained
Δρ_max_ = 0.62 e Å^−3^
Δρ_min_ = −0.31 e Å^−3^

### 

Data collection: *APEX2* (Bruker, 2005 ▶); cell refinement: *SAINT* (Bruker, 2005 ▶); data reduction: *SAINT*; program(s) used to solve structure: *SHELXS97* (Sheldrick, 2008 ▶); program(s) used to refine structure: *SHELXL97* (Sheldrick, 2008 ▶); molecular graphics: *X-SEED* (Barbour, 2001 ▶); software used to prepare material for publication: *publCIF* (Westrip, 2009 ▶).

## Supplementary Material

Crystal structure: contains datablocks global, I. DOI: 10.1107/S160053680903133X/xu2583sup1.cif

Structure factors: contains datablocks I. DOI: 10.1107/S160053680903133X/xu2583Isup2.hkl

Additional supplementary materials:  crystallographic information; 3D view; checkCIF report

## Figures and Tables

**Fig. 1.**
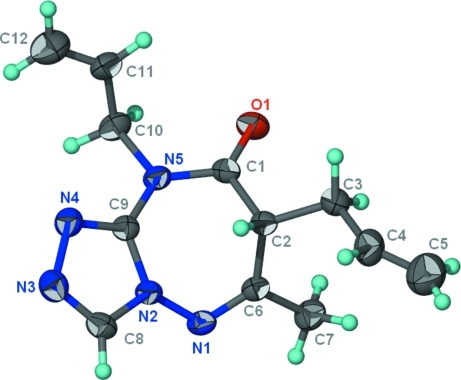
Thermal ellipsoid plot (Barbour, 2001) of C12H15N5O at the 50% probability level; hydrogen atoms are drawn as spheres of arbitrary radius.

## supplementary crystallographic information

### Experimental

To a solution of 6-methyl-7*H*-[1,2,4]triazolo[4,3-*b*][1,2,4]triazepin-8(9*H*)-one (1 g, 6 mmol) in *N*,*N*-dimethylformamide (20 ml), potassium carbonate (1.26 g, 9 mmol), allyl bromide (0.8 ml, 9 mmol) and a catalytic amount of tetrabutyammonium bromide were added. The mixture was stirred for 12 h. After the completion of the reaction (as monitored by TLC), the solid material was removed by filtration and the solvent evaporated under vacuum. Dichloromethane (20 ml) was added and the solution filtered. The solvent was removed and the product purified by column chromatography (30% ethyl acetate/hexane) to afford colorless crystals in 30% yield; m.p. 423 K. The formulation was established by proton and carbon-13 NMR spectroscopy in DMSO-d_6_.

### Refinement

Carbon-bound H-atoms were placed in calculated positions (C—H 0.93 to 0.97 Å) and were included in the refinement in the riding model approximation, with *U*(H) set to 1.2*U*(C).

### Crystal data

**Table:**

C_12_H_15_N_5_O	*F*(000) = 520
*M**_r_* = 245.29	*D*_x_ = 1.299 Mg m^−^^3^
Monoclinic, *P*2_1_/*n*	Mo *K*α radiation, λ = 0.71073 Å
Hall symbol: -P 2yn	Cell parameters from 2666 reflections
*a* = 7.4674 (3) Å	θ = 2.6–23.2°
*b* = 8.3398 (3) Å	µ = 0.09 mm^−^^1^
*c* = 20.2214 (6) Å	*T* = 293 K
β = 95.174 (2)°	Block, colorless
*V* = 1254.19 (8) Å^3^	0.3 × 0.3 × 0.3 mm
*Z* = 4	

### Data collection

**Table:**

Bruker APEXII diffractometer	1600 reflections with *I* > 2σ(*I*)
Radiation source: fine-focus sealed tube	*R*_int_ = 0.041
graphite	θ_max_ = 25.9°, θ_min_ = 2.0°
φ and ω scans	*h* = −9→9
11394 measured reflections	*k* = −10→10
2435 independent reflections	*l* = −22→24

### Refinement

**Table:**

Refinement on *F*^2^	Primary atom site location: structure-invariant direct methods
Least-squares matrix: full	Secondary atom site location: difference Fourier map
*R*[*F*^2^ > 2σ(*F*^2^)] = 0.053	Hydrogen site location: inferred from neighbouring sites
*wR*(*F*^2^) = 0.176	H-atom parameters constrained
*S* = 1.03	*w* = 1/[σ^2^(*F*_o_^2^) + (0.1011*P*)^2^ + 0.1814*P*] where *P* = (*F*_o_^2^ + 2*F*_c_^2^)/3
2435 reflections	(Δ/σ)_max_ = 0.001
164 parameters	Δρ_max_ = 0.62 e Å^−^^3^
0 restraints	Δρ_min_ = −0.31 e Å^−^^3^

### Fractional atomic coordinates and isotropic or equivalent isotropic displacement parameters (Å^2^)

**Table:**

	*x*	*y*	*z*	*U*_iso_*/*U*_eq_	
O1	0.6016 (3)	0.9072 (2)	0.62240 (9)	0.0504 (5)	
N1	0.6575 (3)	0.4291 (2)	0.67475 (9)	0.0372 (5)	
N2	0.6714 (3)	0.4126 (2)	0.60624 (9)	0.0346 (5)	
N3	0.7253 (3)	0.2937 (3)	0.51391 (10)	0.0486 (6)	
N4	0.7390 (3)	0.4593 (3)	0.50437 (9)	0.0424 (6)	
N5	0.7149 (3)	0.6893 (2)	0.57403 (9)	0.0360 (5)	
C1	0.5941 (3)	0.7634 (3)	0.61190 (10)	0.0353 (6)	
C2	0.4588 (3)	0.6533 (3)	0.63974 (10)	0.0328 (6)	
H2	0.4112	0.5834	0.6033	0.039*	
C3	0.3002 (3)	0.7447 (3)	0.66310 (12)	0.0460 (7)	
H3A	0.3402	0.8057	0.7025	0.055*	
H3B	0.2560	0.8200	0.6289	0.055*	
C4	0.1488 (4)	0.6348 (4)	0.67861 (16)	0.0600 (8)	
H4	0.1127	0.5587	0.6465	0.072*	
C5	0.0662 (5)	0.6348 (5)	0.73019 (19)	0.0877 (12)	
H5A	0.0969	0.7084	0.7639	0.105*	
H5B	−0.0256	0.5613	0.7346	0.105*	
C6	0.5616 (3)	0.5471 (3)	0.69075 (10)	0.0338 (6)	
C7	0.5614 (4)	0.5818 (3)	0.76328 (11)	0.0480 (7)	
H7A	0.6349	0.5044	0.7882	0.072*	
H7B	0.4406	0.5759	0.7758	0.072*	
H7C	0.6086	0.6874	0.7724	0.072*	
C8	0.6870 (3)	0.2708 (3)	0.57428 (13)	0.0433 (6)	
H8	0.6722	0.1707	0.5933	0.052*	
C9	0.7056 (3)	0.5264 (3)	0.56022 (11)	0.0334 (6)	
C10	0.8431 (3)	0.7867 (3)	0.53946 (12)	0.0449 (7)	
H10A	0.9421	0.7191	0.5286	0.054*	
H10B	0.8920	0.8704	0.5692	0.054*	
C11	0.7587 (4)	0.8618 (3)	0.47735 (13)	0.0519 (7)	
H11	0.6702	0.9388	0.4813	0.062*	
C12	0.8000 (5)	0.8273 (4)	0.41876 (16)	0.0779 (11)	
H12A	0.8880	0.7509	0.4130	0.093*	
H12B	0.7420	0.8788	0.3820	0.093*	

### Atomic displacement parameters (Å^2^)

**Table:**

	*U*^11^	*U*^22^	*U*^33^	*U*^12^	*U*^13^	*U*^23^
O1	0.0657 (13)	0.0282 (11)	0.0585 (11)	−0.0048 (9)	0.0122 (9)	−0.0007 (8)
N1	0.0448 (12)	0.0352 (12)	0.0313 (10)	−0.0009 (10)	0.0013 (8)	0.0048 (8)
N2	0.0407 (12)	0.0290 (11)	0.0341 (10)	0.0007 (9)	0.0037 (8)	0.0025 (8)
N3	0.0564 (15)	0.0419 (14)	0.0482 (13)	0.0010 (11)	0.0084 (11)	−0.0081 (10)
N4	0.0485 (13)	0.0417 (13)	0.0376 (11)	−0.0001 (10)	0.0073 (9)	−0.0018 (9)
N5	0.0391 (12)	0.0314 (12)	0.0381 (10)	−0.0050 (9)	0.0063 (9)	0.0049 (8)
C1	0.0424 (15)	0.0303 (14)	0.0327 (12)	0.0003 (11)	−0.0005 (10)	0.0010 (10)
C2	0.0363 (13)	0.0296 (13)	0.0324 (12)	−0.0009 (10)	0.0016 (10)	0.0014 (9)
C3	0.0504 (17)	0.0422 (15)	0.0461 (14)	0.0071 (13)	0.0087 (12)	0.0053 (11)
C4	0.0377 (16)	0.081 (2)	0.0619 (18)	0.0146 (15)	0.0103 (13)	0.0091 (16)
C5	0.066 (2)	0.106 (3)	0.093 (3)	0.009 (2)	0.018 (2)	0.018 (2)
C6	0.0360 (14)	0.0328 (13)	0.0323 (12)	−0.0049 (11)	0.0018 (10)	0.0028 (10)
C7	0.0541 (17)	0.0556 (18)	0.0340 (13)	0.0011 (14)	0.0022 (11)	0.0019 (11)
C8	0.0489 (16)	0.0299 (14)	0.0510 (15)	0.0011 (12)	0.0038 (12)	−0.0002 (11)
C9	0.0323 (13)	0.0331 (14)	0.0342 (12)	−0.0008 (10)	0.0004 (9)	0.0016 (10)
C10	0.0443 (16)	0.0436 (16)	0.0475 (14)	−0.0111 (13)	0.0080 (12)	0.0063 (11)
C11	0.0567 (18)	0.0493 (18)	0.0519 (16)	−0.0001 (14)	0.0166 (13)	0.0142 (13)
C12	0.100 (3)	0.079 (3)	0.0560 (19)	0.009 (2)	0.0132 (18)	0.0164 (17)

### Geometric parameters (Å, °)

**Table:**

O1—C1	1.218 (3)	C3—H3B	0.9700
N1—C6	1.276 (3)	C4—C5	1.259 (4)
N1—N2	1.405 (3)	C4—H4	0.9300
N2—C8	1.358 (3)	C5—H5A	0.9300
N2—C9	1.369 (3)	C5—H5B	0.9300
N3—C8	1.293 (3)	C6—C7	1.495 (3)
N3—N4	1.400 (3)	C7—H7A	0.9600
N4—C9	1.304 (3)	C7—H7B	0.9600
N5—C1	1.381 (3)	C7—H7C	0.9600
N5—C9	1.388 (3)	C8—H8	0.9300
N5—C10	1.479 (3)	C10—C11	1.491 (4)
C1—C2	1.511 (3)	C10—H10A	0.9700
C2—C6	1.514 (3)	C10—H10B	0.9700
C2—C3	1.519 (3)	C11—C12	1.283 (4)
C2—H2	0.9800	C11—H11	0.9300
C3—C4	1.510 (4)	C12—H12A	0.9300
C3—H3A	0.9700	C12—H12B	0.9300
			
C6—N1—N2	114.74 (18)	H5A—C5—H5B	120.0
C8—N2—C9	104.5 (2)	N1—C6—C7	116.6 (2)
C8—N2—N1	124.87 (19)	N1—C6—C2	122.7 (2)
C9—N2—N1	129.60 (19)	C7—C6—C2	120.7 (2)
C8—N3—N4	107.47 (19)	C6—C7—H7A	109.5
C9—N4—N3	106.37 (19)	C6—C7—H7B	109.5
C1—N5—C9	121.80 (19)	H7A—C7—H7B	109.5
C1—N5—C10	119.9 (2)	C6—C7—H7C	109.5
C9—N5—C10	117.75 (19)	H7A—C7—H7C	109.5
O1—C1—N5	121.0 (2)	H7B—C7—H7C	109.5
O1—C1—C2	123.7 (2)	N3—C8—N2	110.9 (2)
N5—C1—C2	115.2 (2)	N3—C8—H8	124.5
C1—C2—C6	107.12 (18)	N2—C8—H8	124.5
C1—C2—C3	112.1 (2)	N4—C9—N2	110.7 (2)
C6—C2—C3	116.29 (18)	N4—C9—N5	125.7 (2)
C1—C2—H2	106.9	N2—C9—N5	123.41 (19)
C6—C2—H2	106.9	N5—C10—C11	112.7 (2)
C3—C2—H2	106.9	N5—C10—H10A	109.0
C4—C3—C2	112.3 (2)	C11—C10—H10A	109.0
C4—C3—H3A	109.1	N5—C10—H10B	109.0
C2—C3—H3A	109.1	C11—C10—H10B	109.0
C4—C3—H3B	109.1	H10A—C10—H10B	107.8
C2—C3—H3B	109.1	C12—C11—C10	124.4 (3)
H3A—C3—H3B	107.9	C12—C11—H11	117.8
C5—C4—C3	127.1 (4)	C10—C11—H11	117.8
C5—C4—H4	116.4	C11—C12—H12A	120.0
C3—C4—H4	116.4	C11—C12—H12B	120.0
C4—C5—H5A	120.0	H12A—C12—H12B	120.0
C4—C5—H5B	120.0		
			
C6—N1—N2—C8	−147.4 (2)	C1—C2—C6—C7	101.0 (2)
C6—N1—N2—C9	46.1 (3)	C3—C2—C6—C7	−25.3 (3)
C8—N3—N4—C9	−0.7 (3)	N4—N3—C8—N2	0.8 (3)
C9—N5—C1—O1	−178.4 (2)	C9—N2—C8—N3	−0.6 (3)
C10—N5—C1—O1	−6.7 (3)	N1—N2—C8—N3	−170.0 (2)
C9—N5—C1—C2	3.0 (3)	N3—N4—C9—N2	0.3 (3)
C10—N5—C1—C2	174.68 (19)	N3—N4—C9—N5	176.1 (2)
O1—C1—C2—C6	−110.7 (2)	C8—N2—C9—N4	0.2 (3)
N5—C1—C2—C6	67.9 (2)	N1—N2—C9—N4	168.8 (2)
O1—C1—C2—C3	18.0 (3)	C8—N2—C9—N5	−175.7 (2)
N5—C1—C2—C3	−163.40 (19)	N1—N2—C9—N5	−7.1 (4)
C1—C2—C3—C4	169.1 (2)	C1—N5—C9—N4	142.8 (2)
C6—C2—C3—C4	−67.2 (3)	C10—N5—C9—N4	−29.0 (3)
C2—C3—C4—C5	130.9 (3)	C1—N5—C9—N2	−41.9 (3)
N2—N1—C6—C7	−172.6 (2)	C10—N5—C9—N2	146.2 (2)
N2—N1—C6—C2	4.6 (3)	C1—N5—C10—C11	−78.4 (3)
C1—C2—C6—N1	−76.1 (3)	C9—N5—C10—C11	93.6 (3)
C3—C2—C6—N1	157.6 (2)	N5—C10—C11—C12	−114.4 (3)
